# Cut-off scores for mild and moderate dementia on the Addenbrooke's Cognitive Examination-III and the Mini-Addenbrooke's Cognitive Examination compared with the Mini-Mental State Examination

**DOI:** 10.1192/bjb.2023.27

**Published:** 2024-02

**Authors:** Louise McCarthy, Judy Rubinsztein, Ellen Lowry, Emma Flanagan, Vandana Menon, Silvia Vearncombe, Eneida Mioshi, Michael Hornberger

**Affiliations:** 1 Norfolk and Suffolk NHS Foundation Trust, Norwich, UK; 2 Cambridge and Peterborough NHS Foundation Trust, Cambridge, UK; 3 Norwich Medical School, University of East Anglia, Norwich, UK; 4 School of Health Sciences, University of East Anglia, Norwich, UK

**Keywords:** Carers, community mental health teams, dementia, rating scales, psychological testing

## Abstract

**Aims and method:**

We aimed to establish cut-off scores to stage dementia on the Addenbrooke's Cognitive Examination-III (ACE-III) and the Mini-Addenbrooke's Cognitive Examination (M-ACE) compared with scores traditionally used with the Mini-Mental State Examination (MMSE). Our cross-sectional study recruited 80 patients and carers from secondary care services in the UK.

**Results:**

A score ≤76 on the ACE-III and ≤19 on the M-ACE correlated well with MMSE cut-offs for mild dementia, with a good fit on the receiver operating characteristic analysis for both the ACE-III and M-ACE. The cut-off for moderate dementia had lower sensitivity and specificity. There were low to moderate correlations between the cognitive scales and scales for everyday functioning and behaviour.

**Clinical implications:**

Our findings allow an objective interpretation of scores on the ACE-III and the M-ACE relative to the MMSE, which may be helpful for clinical services and research trials.

A diagnosis of dementia is usually made following specialist review that includes a clinical history and examination involving patients and carers, a dementia blood screen, cognitive assessment, assessment of activities of daily living, behavioural problems and sometimes brain imaging.^1,2^ Although the Mini-Mental State Examination (MMSE) is still the gold standard tool employed in research and clinical trials, the Addenbrooke's Cognitive Examination-III (ACE-III) screening tool is increasingly used in UK memory clinics as an alternative to the MMSE. It is a practical bedside test, easy to use and freely accessible to all, unlike the MMSE and Montreal Cognitive Assessment (MoCA), which are both subject to copyright agreements.^3,4^ A shorter version, the Mini-Addenbrooke's Cognitive Examination (M-ACE), has also been developed and is considered helpful where patients struggle with longer cognitive examinations; the M-ACE has validated cut-offs of ≤25/30 and ≤21/30 in screening for dementia versus no dementia.^5^

There is inconsistent evidence on how to categorise dementia severity. Clinicians and researchers have often used the MMSE to help them in defining clinical staging, with the following recommended ranges and cut-offs: mild: 21–26 (cut-off ≤26); moderate: 10–20 (cut-off <21); severe: <10.^2^ We examined similar guiding cut-off scores to stage dementia using the ACE-III and M-ACE. We also examined how cognition correlated with everyday functioning and neuropsychiatric questionnaires.

## Method

We used a cross-sectional study design.

### Participants and data collection

Patients were identified by clinicians in secondary mental health services. We included patients over 50 years of age with a clinician-diagnosed dementia (all subtypes and severity) who were sufficiently clinically stable to undertake the assessment. Patients had to have a study partner (‘carer’, defined as someone who knew the person well, had contact with them for at least 1 h per week) and were happy to complete informed consent and answer questions about the person's functional abilities. All patients and carers received separate study information sheets and signed consent forms before any study activity took place. A mental capacity assessment was undertaken as part of the informed consent process. Patients lacking capacity were included and we used a separate consultee assent process for these individuals.

All study procedures comply with the ethical standards of relevant national and institutional committees on human experimentation and with the Helsinki Declaration of 1975, as revised in 2008. The study took place within the demenTia Research and Care Clinic (TRACC) study at the University of East Anglia (IRAS ID: 205788) with ethics approval.

### Description of instruments

#### Global cognition

The Addenbrooke's Cognitive Examination-III (ACE-III) is a 26-item cognitive scale measuring the following domains: attention, memory, language, fluency and visuospatial. A higher total suggests less cognitive impairment, with a total possible score of 100.^6^

The Mini-Addenbrooke's Cognitive Examination (M-ACE), which is derived from the ACE-III, assesses the attention, memory, verbal fluency and visuospatial (clock drawing) domains.^5^ A higher total suggests less cognitive impairment, with a total possible score of 30.

The Mini-Mental State Examination (MMSE) is a brief 30-item screening tool assessing domains similar to those in the ACE-III and M-ACE, with a higher total indicating less cognitive impairment.^3^ Traditionally used widely in clinical practice, its clinical utility is now affected by being subject to copyright. The copyright version (the MMSE-2)^7^ was used for our study with permission of PAR (parinc.com). The MMSE2 is analogous to the original and, in the interest of ease of reading, is referred to as the MMSE throughout this paper.

#### Behaviour and everyday function

The Cambridge Behavioural Inventory-Revised (CBI-R) is a 45-item scale on which carers are asked to rate the frequency (never to constantly) of changes, cognitive and behavioural (i.e. memory and orientation, everyday skills, self-care), observed over the previous month.^8^

The Frontotemporal Dementia Rating Scale (FTD-FRS) is a novel tool assessing neuropsychiatric symptoms (i.e. lack of affection, impulsivity) and everyday functioning (e.g. going out, shopping, household chores, using a telephone, taking medications). The tool has previously been used in determining disease progression and severity stage in the subtype frontotemporal dementia; it is also useful in other dementia subtypes and the semi-structured interview format was considered useful to complement the CBI-R for the purposes of our study.^9^

### Statistical analysis

Data were analysed using SPSS Statistics 25 for Windows.^10^ Before any analysis, variables were plotted and checked for normality of distribution using the Shapiro–Wilk test. Pearson correlations were conducted to investigate relationships between the total scores on the three scales included in this analysis (ACE-III, M-ACE, MMSE).

MMSE scores for mild, moderate and severe dementia were stratified using National Institute for Health and Care (NICE) guidelines (mild: MMSE score ≤26; moderate: <20; severe: <10).^2^ Scatterplots were calculated to compare the raw scores between the MMSE and the ACE-III and the MMSE and the M-ACE. This exploration was based on similar conversion analyses.^11^ Corresponding conversion scores were calculated for the ACE-III and M-ACE using a regression score derived from the linear relationship (*y* = *a* + *bx*, where *x* is the independent variable, *y* is the dependent variable, *b* is the slope and *a* is the *y*-intercept) between the scales from these scatterplots. This created binary variables for logistic regression, which was used to model the probability of mild or moderate cut-offs. Receiver operating characteristic (ROC) analysis was employed to measure the accuracy of the different cut-offs (mild and moderate) on the ACE-III compared with the MMSE and on the M-ACE compared with the MMSE. There was further exploration, using ROC analysis, of the three cognitive scales and the CBI-R and FTD-FRS.

## Results

### Participants

We recruited 80 patients and their carers over a 2-year period from March 2017 to April 2019. All were of White British or Irish ethnicity, with English as their first language. The majority of participants were educated to secondary school level (60%), with a small proportion (23%) receiving further education. Alzheimer's dementia was the most common diagnosis, followed by dementia with Lewy bodies (DLB), mixed dementia and vascular dementia; one individual had a diagnosis of Parkinson's disease dementia. Mean scores on the three cognitive instruments were: MMSE, 20.7 (s.d. = 5.7); ACE-III, 63.7 (s.d. = 15.7); and M-ACE, 12.5 (s.d. = 6.3).

Of the 80 participants recruited into the study, 15 were removed from the ACE-III analysis and 9 were removed from the M-ACE analysis because of missing data.

### Relationship of MMSE scores with ACE-III and M-ACE scores

Regression equations for determining ACE-III and M-ACE cut-offs based on the MMSE NICE guideline cut-offs were based on the linear relationship between these scales using a scatterplot.

The regression equations were:
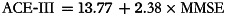

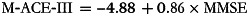


**Fig. 1:**
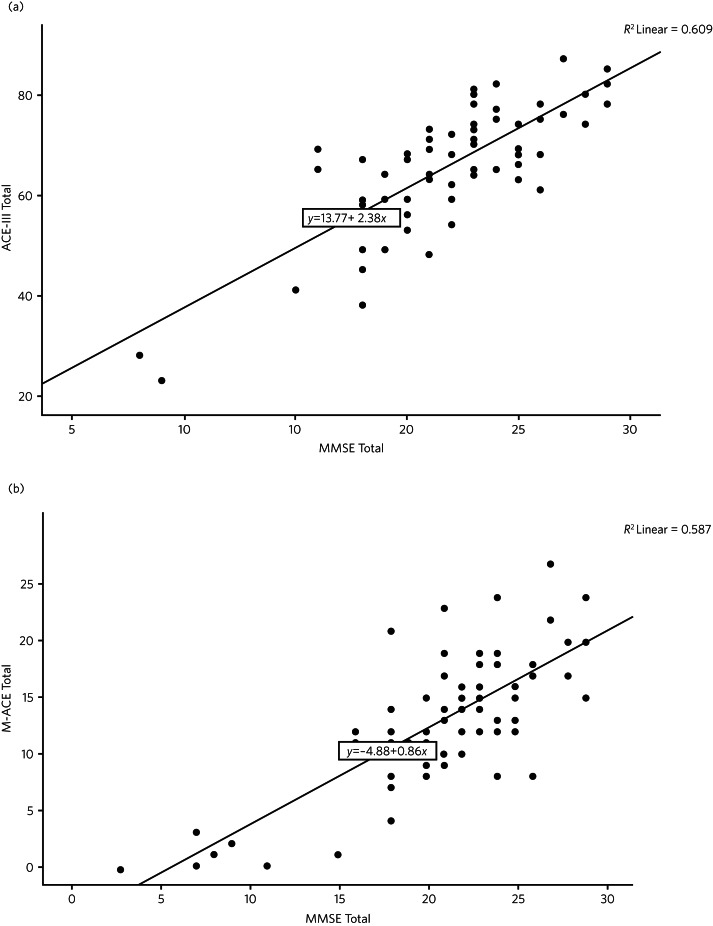
(a) Scatterplots of raw scores for the Mini-Mental State Examination (MMSE) and Addenbrooke's Cognitive Examination-III (ACE-III). (b) Scatterplot of raw scores for the MMSE and the Mini-Addenbrooke's Cognitive Examination (M-ACE).

The scatterplot in Fig. 1 shows the relationship between raw scores on the MMSE and the ACE-III and on the MMSE and the M-ACE.

Examining the raw scores, a score of 76/100 on the ACE-III approximately equates to the highest ‘mild dementia’ score on the MMSE (26), a score of 59/100 on the ACE-III equates with the top of the moderate range on the MMSE (<20). The Pearson correlation coefficient between the ACE-III and the MMSE total scores was 0.78 (*P* = 0.001).

For the M-ACE according to the raw scores, a score of 19/30 on the M-ACE is approximately equal to or less than 26 (mild) on the MMSE, a total M-ACE score of 13/30 is approximately equal to or less than 20 (moderate) on the MMSE. The Pearson correlation coefficient between M-ACE and MMSE total scores was 0.77 (*P* = 0.001).

There were insufficient data on both scales to calculate meaningful scores for severe dementia.

### Sensitivity and specificity analysis

Sensitivity and specificity analyses were performed using logistic regression and ROC analysis to determine the effectiveness of the ACE-III and M-ACE cut-off scores derived from the MMSE NICE cut-off scores using the regression equations determined above. Mild and moderate groups determined by these new cut-off scores were explored using MMSE performance.

#### ACE-III

Twelve patients were removed from the analysis exploring ACE-III mild and moderate groups because they scored above the threshold for the mild group, as determined by the conversion analysis above based on the MMSE (ACE-III score <76). Logistic regression for the mild and moderate groups determined by the new ACE-III cut-offs indicated that the regression model based on the MMSE score predictor was statistically significant (χ^2^(1) = 18.86, *P* < 0.001). The model explained 42.4% (Nagelkerke *R*^2^) of the variance in ACE-III group severity and correctly classified 77.4% of participants with mild and moderate dementia scores (33/37 (89.2%) with mild; 8/16 (50.0%) with moderate) into their respective cohorts.

**Fig. 2:**
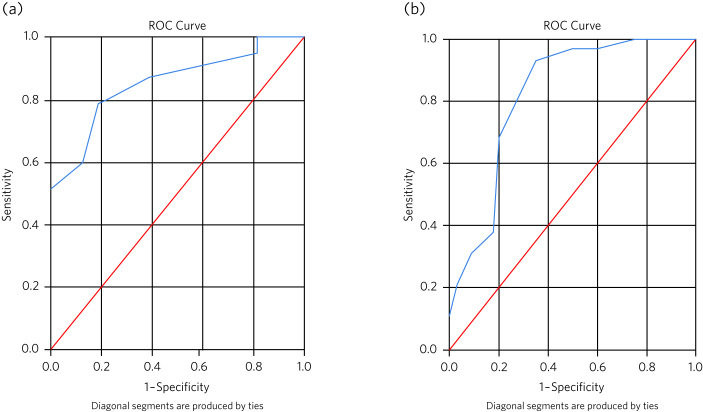
(a) The receiver operating characteristic (ROC) curves for the Addenbrooke's Cognitive Examination-III (ACE-III) with a cut-off of 76 (mild dementia) when associated with the Mini-Mental State Examination (MMSE). (b) The receiver operating characteristic curves for the Mini-Addenbrooke's Cognitive Examination (M-ACE) with a cut-off of 19 (mild dementia) when associated with the MMSE.

Figure 2(a) shows the ROC analysis for mild dementia on the ACE-III according to scores on the MMSE. For an ACE-III cut-off for mild dementia of 76/100, area under the curve analysis (AUC) was estimated at 0.85 (s.e. = 0.05, 95% CI 0.75–0.95), which is significantly different from AUC = 0.5 (indicating no discrimination at *P* < 0.001). For a cut-off for moderate dementia of 59/100, the AUC was estimated at 0.15 (s.e. = 0.05, 95% CI 0.05–0.25), which showed poor sensitivity and specificity to detect the moderate disease stage (Supplementary Fig. 3(a) available at https://doi.org/10.1192/bjb.2023.27).

#### M-ACE

Eight patients were removed from the analysis of M-ACE groups based on the new cut-offs because they scored above the upper cut-off for the mild group, as determined by the conversion analysis above based on the MMSE (M-ACE score <19). Logistic regression indicated that the regression model based on the MMSE total score predictor was statistically significant (χ^2^(1) = 24.01, *P* < 0.001). The model explained 42.3% (Nagelkerke *R*^2^) of the variance in M-ACE group severity and correctly classified 76.2% of participants with mild and moderate dementia scores (2/29 (79.3%) with mild; 25/34 (76.2%) with moderate) into their respective cohorts.

Figure 2(b) shows the ROC analysis for mild dementia on the M-ACE according to scores on the MMSE. For a M-ACE cut-off for mild dementia of 19/30, the AUC was estimated at 0.81 (s.e. = 0.05, 95% CI 0.71–0.93), which is significantly different from AUC = 0.5 (*P* < 0.001). For a cut-off for moderate dementia of 13/30, the AUC was estimated at 0.18 (s.e. = 0.05, 95% CI 0.08–0.29), which showed poor sensitivity and specificity to detect the moderate disease stage (Supplementary Fig. 3(b)).

#### Functional performance at ACE-III and M-ACE cut-offs

There were low to moderate negative correlations between the cognitive scales and the CBI-R and the ACE-III (*r* = −0.24, *P* = 0.05), M-ACE (*r* = −0.29, *P* = 010) and MMSE (*r* = −0.34, *P* = 0.005) and low to moderate positive correlations between the FTD-FRS logit score and the ACE-III (*r* = 0.35, *P* = 0.003), M-ACE (*r* = 0.38, *P* = 0.001) and MMSE (*r* = 0.47, *P* < 0.001). Logistic regression and ROC analysis were not significant at the ACE-III- and M-ACE-derived mild and moderate cut-offs for both functional scales.

## Discussion

We found that a score ≤76 on the ACE-III and ≤19 on the M-ACE correlated well with MMSE cut-offs for mild dementia, with a good fit on the ROC analysis for both the ACE-III and the M-ACE. However, with moderate dementia, examining the scatterplots of the raw scores for the MMSE against the ACE-III and the M-ACE (Supplementary Fig. 3(a) and (b)) there is a lot of scatter around moderate cut-off points on the MMSE, suggesting that it will be inherently difficult to compare cut-off points at this stage of disease using the MMSE versus the ACE-III and M-ACE. The ROC analysis showed a poor fit in this model in the moderate range for both the ACE-III and the M-ACE and was not sensitive or specific in moderate dementia stages. This may be due, in part, to problems in performing cognitive testing in patients with more severe symptoms, where some individuals may not be able to cope with completing the cognitive scales at all or may be able to tolerate only partial completion. Therefore we recommend that cut-offs suggested for the moderate dementia stage in this study should be interpreted and used with caution.

### Comparison with the literature

A Cochrane review highlights an overall lack of evidence on the detection of dementia using the ACE-III and M-ACE. The review authors concluded that there was insufficient evidence to recommend the ACE-III and M-ACE for screening dementia and mild cognitive impairment. Interestingly, they found a lack of evidence of efficacy in primary care but found that these tools may be useful in secondary services when, using the lower thresholds of 82 for the ACE-III and 21 for the M-ACE, fewer false-positive dementia diagnoses (versus no dementia) were reported.^12^ Additionally, evidence suggests that the optimal cut-offs for distinguishing dementia from mild cognitive impairment using the M-ACE are one point lower than those reported in the original validation study for both the high sensitivity (≤24/30 *v*. ≤25/30) and high specificity (≤20/30 *v*. ≤21/30) cut-offs.^13^ Another study suggested that an ACE-III cut-off of 61 may be helpful in differentiating between mild and moderate dementia, but results were regarded as exploratory as the number of patients included was small.^14^ Our study, as well as consolidating previous ACE-III findings, provides, for the first time, cut-offs for the M-ACE for mild and moderate dementia. This is important as the M-ACE is now used routinely in clinical practice. We have established cut-offs to be used as a guide, clinically and in research, to define mild and moderate dementia, by comparing with the established MMSE cut-offs.

### Cut-offs for severe dementia

Owing to the small number of patients with severe dementia, we were not able to robustly establish the cut-offs for the severe stage on either the ACE -III or the M-ACE. It is particularly challenging to recruit individuals with severe dementia to research studies and they can find it difficult to complete cognitive rating scales. Arguably, in clinical practice it is easier to classify the advanced/severe dementia stage as these patients may be unable to complete cognitive scales and have lost many functional skills. This emphasises the need for clinicians to combine clinical judgement with interpretation of rating scale scores in dementia diagnosis and staging.

### Correlations between cognition, behaviour and functioning

Additionally, we explored correlations between patients’ cognition and their behaviour and functioning using two functional scales: the CBI-R (self-completed) and the FTD-FRS (semi-structured interview). Both completed by the carer, the self-completion aspect of the CBI-R may facilitate honest expression and the interview process of the FTD-FRS may encourage open reflection. It may be that using a combination of both scales will be of benefit in a clinical assessment. The low to moderate correlations between the cognitive scales (ACE-III and M-ACE) and the functional scales (CBI-R and FTD-FRS) are to be expected and replicate previous findings.^6^ Cognitive scales have an emphasis on cognition and language, whereas the functional scales assess behaviour and function; therefore a high score on a cognitive scale does necessarily exclude moderate or severe dementia. It is recognised that functional impairments are linked to cognitive deficits, but precise understanding of this complex link is lacking.^8,14^

Combining a cognitive tool with a functional tool is likely to produce a more accurate picture of dementia severity. A clinician will interpretate the cognitive score and the functional abilities of the patient in order to stage the severity of a dementia. This process is important as it allows patients, carers and clinicians to consider the most appropriate treatment options and it informs decision-making and advance planning.^2^ Our findings add to the current body of evidence and are in accordance with NICE guidance and recent Cochrane evidence.^2,12^

### Patient sample

The sample of patients engaged in our study is very typical of those seen in old age psychiatric memory clinics in the UK in terms of diagnostic range and age.^1,15^ The DLB subtype was the second most common diagnosis, which may have been raised in our sample as we were a recruiting site for the DIAMOND-Lewy study, which focuses on identification of DLB using a toolkit.^16^ The mean age was 78 years (range 58–90), with 46% (*n* = 37) in the older old age range (80–90 years). Increasing age is the main risk factor in the development, and in the worsening, of dementia, and the average age of 78 in our sample may partly explain the lower ACE-III cut-off of 76 in the mild group found in our study compared with the previous evidence^6^ (where the commonly employed ACE-III cut-off to delineate mild dementia was below 82). Our study was also not a comparison study against normal controls as in the previous work.^6^ The cut-off for moderate dementia in our study is lower than in previous evidence,^14^ which may be explained by the larger sample size, more severely affected patients and the slightly older group of patients in our study. Furthermore, the inclusion of participants who lacked capacity in our study adds to the overall practical clinical value of our findings.

### Limitations

Despite our important findings, there are certain limitations to our study. Our sample included patients at all stages of disease severity, resulting in missing data as some individuals were unable to complete all the rating scales. More spread of data across the moderate to severe stages would have added to the analysis and interpretation.

Our sample, although representative of the local population in our memory clinics in East Anglia, did not include any individuals from an ethnic minority background. Additionally, English was the first language of all participants. We acknowledge the inherent cultural bias in all cognitive tests and recommend that our findings are validated in other populations.

Finally, our findings cannot be generalised beyond the four dementia subtypes included in our study. The absence of any patients in our sample with behavioural or language-variant frontotemporal dementia means that the correlations between cognitive instruments shown here cannot be presumed to apply in these disorders.^6^ In addition, DLB is overrepresented in our cohort compared with typical English memory service populations.^17^ Future studies should examine cut-off scores by dementia subtype to better define the performance of the ACE-III and M-ACE in different dementia syndromes.

**Table 1:** Demographics for the patient sample (*n* = 80)

Characteristic	
Age, mean (s.d.)	78.23 (7.11)
Gender (male/female), *n*	55/25
Completed education, *n*	
Primary	2
Secondary	48
Further	14
Higher	5
Missing data	11
Time since diagnosis, *n*	
<6 months	36
6–12 months	13
1–2 years	14
3–5 years	11
>5 years	6
Cholinesterase inhibitor use	78%
Statin use	44%
Diagnosis, *n*	
Alzheimer's dementia	40
Dementia with Lewy bodies	15
Mixed dementia	14
Vascular dementia	10
Parkinson's disease dementia	1
Neuropsychiatric assessment, mean (s.d.)	
MMSE score	20.81 (5.71)
ACE-III score	63.78 (15.70)
M-ACE score	12.58 (6.33)

MMSE, Mini-Mental State Examination; ACE-III, Addenbrooke's Cognitive Examination-III; M-ACE, Mini-Addenbrooke's Cognitive Examination.

## About the authors

**Louise McCarthy** is a senior nursing research lead with the Research and Development Team at Norfolk and Suffolk NHS Foundation Trust, Norwich, UK. **Judy Rubinsztein** is a consultant psychiatrist with Cambridge and Peterborough NHS Foundation Trust (formerly Norfolk and Suffolk NHS Foundation Trust), Cambridge, UK. **Ellen Lowry** is a lecturer in medical education at Norwich Medical School, University of East Anglia, Norwich, UK. **Emma Flanagan** is a clinical trials manager at Norwich Medical School, University of East Anglia, Norwich, UK. **Vandana Menon** is a consultant psychiatrist with Cambridge and Peterborough NHS Foundation Trust (formerly Norfolk and Suffolk NHS Foundation Trust), Cambridge, UK. **Silvia Vearncombe** is a consultant psychiatrist with Cambridge and Peterborough NHS Foundation Trust (formerly Norfolk and Suffolk NHS Foundation Trust), Cambridge, UK. **Eneida Mioshi** is Professor in Dementia Care Research in the School of Health Sciences, University of East Anglia, Norwich, UK. **Michael Hornberger** is Professor of Applied Dementia Research at Norwich Medical School, University of East Anglia, Norwich, UK.

## Supporting information

McCarthy et al. supplementary material McCarthy et al. supplementary material

## Data Availability

Data are available from the corresponding author, L.M., on reasonable request.
