# Supplementary material for: Cut-off scores for mild and moderate dementia on the Addenbrooke's Cognitive Examination-III and the Mini-Addenbrooke's Cognitive Examination compared with the Mini-Mental State Examination
Source: BJPsych Bull. 2024 Feb;48(1):12–8. doi: 10.1192/bjb.2023.27 (PMC10801363; doi:10.1192/bjb.2023.27)

*Figure 3 A. and B*

*A.* ﻿The receiver operating characteristic curves for the ACE- III with a cut-off of 59 (moderate) when associated with the MMSE.

*B.* The receiver operating characteristic curves for the M-ACE with a cut-off of 13 (moderate) when associated with the MMSE.

A. B.

B
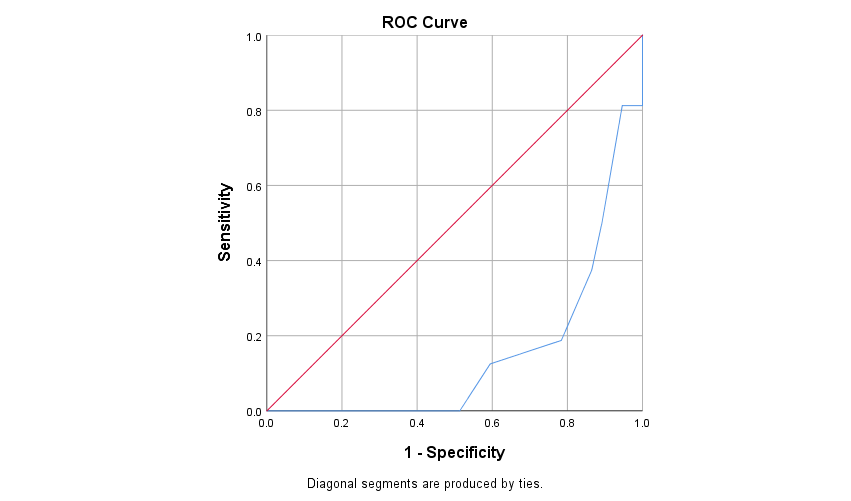

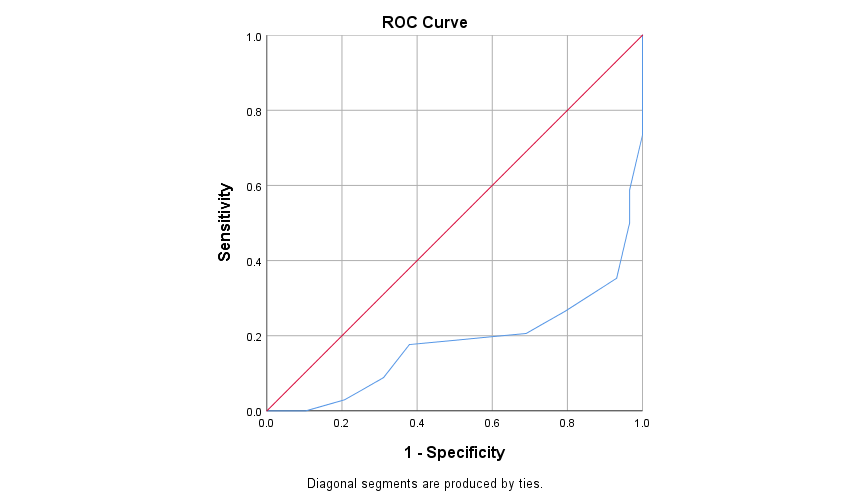

Supplement: McCarthy et al. supplementary material [file S205646942300027Xsup001.docx]
